# HEALTH SYSTEM IMPLEMENTATION, SCALING, AND IMPACT OF THE 4MS FRAMEWORK TO ADVANCE AGE-FRIENDLY CARE

**DOI:** 10.1093/geroni/igac059.2038

**Published:** 2022-12-20

**Authors:** Julia Adler-Milstein, Courtney Lyles, Stephanie Rogers

**Affiliations:** University of California, San Francisco, San Francisco, California, United State; University of California, San Francisco, San Francisco, California, United State; University of California, San Francisco, San Francisco, California, United State

## Abstract

There is a critical need to redesign the healthcare system to provide more effective and tailored care to older adults. The 4Ms Framework (What Matters, Medication, Mentation and Mobility) offers a blueprint to guide health system efforts to deliver more age-friendly care. We sought to characterize and assess real-world implementation experiences with the 4Ms in three health systems (University of California, San Francisco, University of Utah, & Anne Arundel Medical Center). Specifically, we conducted semi-structured interviews (N=29) with diverse stakeholders from each site to characterize the approach to operationalize the framework and assess the implementation experience. Via cross-site content analysis of interview transcripts, we identified four themes. First, the 4Ms offer a compelling conceptual framework around which to organize health system efforts to advance age-friendly care, but each “M” required distinct, complex implementation work that resulted in a fragmented implementation experience. Second and relatedly, inpatient and ambulatory efforts to implement the 4Ms became disconnected, with missed opportunities for synergies and gave little attention to the 4Ms during care transitions. Third, non-physician leadership was key to initiating 4Ms implementation efforts, but obtaining physician buy-in, which often came later, was essential to full implementation. Fourth, sustained 4Ms implementation efforts required both top-down vision and communication from leadership as well as bottom-up culture change that engaged and motivated frontline workers. As efforts to spread the 4Ms Framework advance, these results offer important implementation guidance and also suggest domains in which the Framework may need to be adapted to accommodate real-world implementation experiences.

